# Persistent Left Superior Vena Cava: Implications for Long-Term Arteriovenous Fistula Patency and Potential Cardiac Complications

**DOI:** 10.7759/cureus.90296

**Published:** 2025-08-17

**Authors:** Christopher D Louviere, Alejandro Serrano-Berrios, Dheeraj R Gopireddy, Taylor S Harmon, Grit A Adler, Renato Abu Hana

**Affiliations:** 1 Radiology, University of Florida College of Medicine – Jacksonville, Jacksonville, USA

**Keywords:** arteriovenous fistula, chronic kidney disease, hemodialysis, persistent left superior vena cava, vascular anomaly

## Abstract

A persistent left superior vena cava (PLSVC) is a rare congenital thoracic venous anomaly where the left SVC exists as a separate vessel and drains into the right atrium (RA), most commonly, through the coronary sinus (CS) in about 80-90% or directly into the left atrium (LA). This vascular abnormality can present challenges during central dialysis catheter placement and for the maintenance of arteriovenous fistulas (AVF). In addition, dilation of the coronary sinus from increased blood flow secondary to a PLSVC can cause cardiac complications, especially arrhythmias. We present a case of PLSVE incidentally detected after a dysfunctional arteriovenous fistula.

## Introduction

A persistent left superior vena cava (PLSVC) is a rare congenital thoracic venous anomaly where the left SVC exists as a separate vessel and drains into the right atrium (RA), most commonly through the coronary sinus (CS), in about 80-90% of cases, or directly into the left atrium (LA) [1,2]. Normally, the superior vena cava (SVC) is present in the right thorax and returns blood from the head, neck, and arms to the right atrium at the level of the third costal cartilage. A PLSVC originates embryologically through failure of the left anterior cardinal vein to naturally regress. 

A PLSVC can present challenges to patient health and intervention, especially during central dialysis catheter placement and for the maintenance of arteriovenous fistulas (AVFs). Dilation of the coronary sinus from increased blood flow secondary to a PLSVC can cause cardiac complications, especially arrhythmias. Obstructed venous return can devolve into superior vena cava syndrome and possible cyanosis. 

We present a case of PLSVC incidentally detected after a dysfunctional arteriovenous fistula and describe implications on future management of our patient. 

## Case presentation

A 75-year-old male with end-stage renal disease (ESRD) and a left brachial-basilic AVF created in November 2022 at an outside institution presented to the emergency department after a failed hemodialysis session. Doppler ultrasound demonstrated partial thrombosis of the fistula. The patient was admitted, and the interventional radiology department was consulted for a fistulogram and fistula declotting. During clinical evaluation, the patient was also found to have atrial fibrillation (AF), which had been diagnosed after the AVF creation.

**Figure 1:**
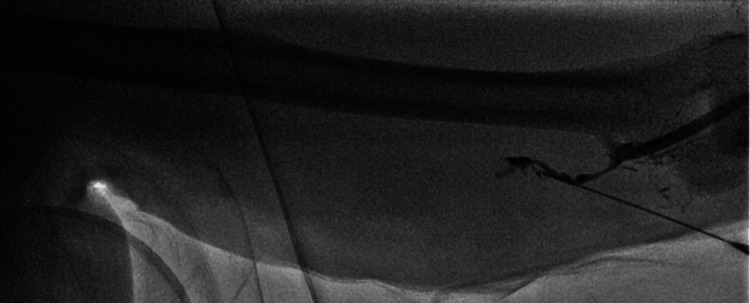
Initial fistulogram performed close to the arteriovenous anastomosis showing atretic anastomosis.

**Figure 2:**
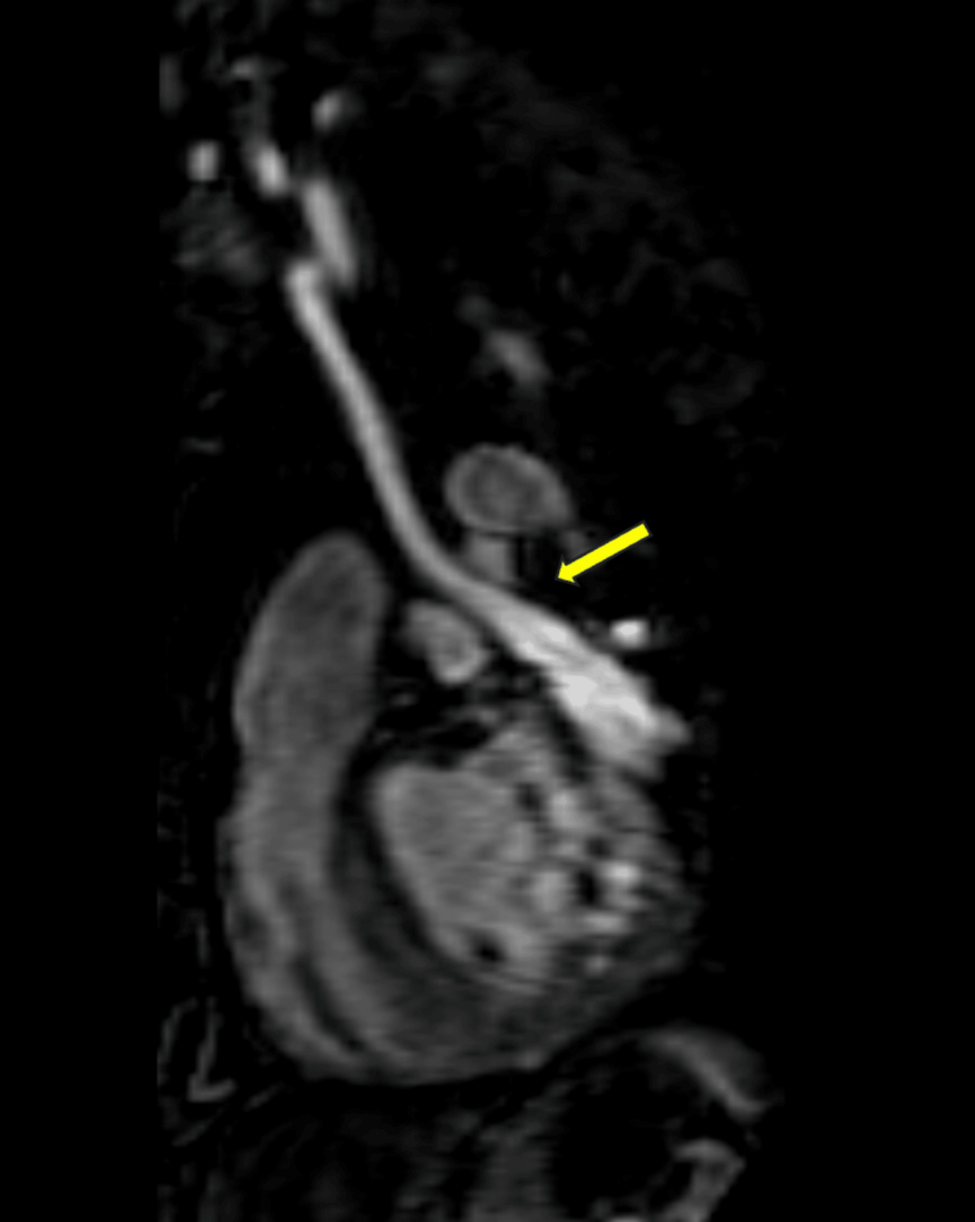
MRA chest reformat showing the PLSVC draining into the mildly dilated coronary sinus (yellow arrow). MRA: magnetic resonance angiography, PLSVC: persistent left superior vena cava

**Figure 3:**
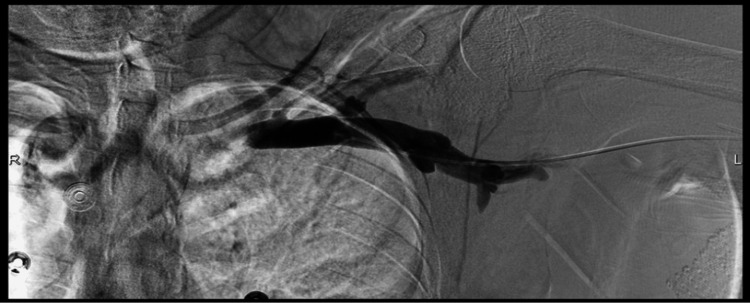
Central venogram showing slow flow throughout the left subclavian vein and the PLSVC. Unfortunately, due to a technical issue, the venogram of the PLSVC draining into the coronary sinus was not recorded. PLSVC: persistent left superior vena cava

The declotting procedure was performed successfully, followed by a fistulogram that demonstrated a persistent left SVC (Figure 1), which was unknown to our team until the time of the procedure. We noticed atretic AV anastomosis along with venous anomalies involving the termination of the left subclavian vein (Figure 2). Hence, a decision was made to place a tunneled catheter via the right internal jugular vein (Figure 3). 

Given the rare incidental finding, we requested a chest and cardiac magnetic resonance imaging (MRI) (Figure 3) with velocity flow mapping. The study not only confirmed the presence of a persistent left SVC but also revealed an incomplete double aortic arch- another vascular anomaly. The velocity flow study demonstrated slower flow from the left SVC into the coronary sinus compared to the normal right SVC into the right atrium (Tables 1, 2), which we believe may interfere with fistula maturation and long-term patency.

**Table 1:** Left SVC data showing decrease in PLSVC flow SVC: superior vena cava, PLSVC: persistent left superior vena cava

Region	ROI 3_H40.6
Flow	
Avg flow over range	-15.694 mL/s
Avg flow per minute	-0.901 L/min
Peak flow	-24.244 mL/s
Time to peak flow	173.667 ms
Forward volume	0 mL
Reverse volume	15.809 mL
Net forward volume	-15.809 mL
Regurgitant fraction	------ %
Body surface area	1.966 m2
Avg flow per minute/BSA	-0.458 L/min/m2
Net forward volume/BSA	-8.042 mL/m2

**Table 2:** Left SVC data showing decrease in PLSVC velocity SVC: superior vena cava, PLSVC: persistent left superior vena cava

Region	ROI 3_H40.6
Velocity and pressure	
Average velocity	-12.682 cm/s
Peak velocity	-36.768 cm/s
Time to peak velocity	208.4 ms
Peak average velocity	-19.591 cm/s
Time to peak average velocity	173.667 ms
Pressure gradient (4vmax2)	0.541 mmHg
Area	
Average area	1.238 cm2
Minimal area	1.238 cm2
Maximal area	1.238 cm2

## Discussion

A persistent SVC is the most common congenital vascular abnormality within the thoracic venous system, occurring in 0.3-0.5% of the population [1,2]. It typically results from the failure of the left anterior and common cardinal vein to regress during embryologic development [1-3].

Unlike the typical right-sided SVC draining into the right atrium, a PLSVC commonly empties into the coronary sinus (CS), which normally handles only about 20% of total venous return [4,5]. While PLSVC is typically asymptomatic, the creation of an arteriovenous fistula (AVF) in the left upper extremity can significantly alter venous return dynamics by redirecting a substantial volume of blood into the CS. As the CS accommodates the increased volume, it may undergo progressive dilation, potentially reaching aneurysmal proportions. When significant, this dilation can cause a mass effect on adjacent conduction system structures, including the sinus/atrioventricular (AV) node and His bundle, and trigger arrhythmias, such as atrial fibrillation (AF), atrial flutter, and ventricular fibrillation [6-10].

Presumably, left-sided AVF can aggravate CS dilatation caused by volume overload due to draining of the PLSVC into the CS. In addition, increased venous turbulence and pressure may contribute to intimal hyperplasia, predisposing the fistula to stenosis or failure to mature adequately, as seen in this case report.

In this reported patient, MRI cardiac assessment revealed that although there is an increased blood flow into CS via PLSVC (approximately 15 mL/s), it is still reduced when compared to the average normal right SVC draining into the right atrium (23-40 mL) [1,4]. This imbalance may compromise AVF maturation and threaten long-term patency.

Unfortunately, we found only five case reports describing PLSVC coexisting with a left upper extremity dialysis arteriovenous fistula in the literature. In all cases, the PLSVC was discovered incidentally, as in the presented case. In two cases, the AV fistula was ligated, in two cases, there was no reported data, and in one case, the AV fistula was maintained [6,9,10,11,12].

PLSVC has been implicated in both the initiation and maintenance of AF, and in our case, the patient’s history of non-sustained arrhythmias, along with imaging evidence of a mildly enlarged CS, strongly suggests AV node compression as a plausible mechanism [5,11].

This constellation of risks underscores the importance of close clinical monitoring, including serial imaging to assess CS size, rhythm surveillance to detect arrhythmias, and functional evaluation of AVF performance over time. This case highlights the importance of performing central venous mapping, in addition to the routinely conducted peripheral arterial and venous Doppler ultrasound, to evaluate vascular anatomy prior to AV fistula creation.

## Conclusions

PLSVC can have important clinical implications when unrecognized, particularly in patients requiring hemodialysis access. In this case, PLSVC associated with a left upper extremity AVF likely contributed to inadequate fistula maturation, subsequent thrombosis, coronary sinus dilation, and new-onset arrhythmias. Although uncommon, PLSVC should be identified through pre-procedural central venous mapping, as its presence represents at least a relative contraindication to left-sided AVF creation. When an AVF is placed in this context, careful monitoring of fistula maturation, long-term patency, and cardiac status is essential.
